# 1577. HIV Viral Load Suppression in Patients Utilizing an Integrated Health System Specialty Pharmacy Compared to a Non-integrated Specialty Pharmacy Model

**DOI:** 10.1093/ofid/ofad500.1412

**Published:** 2023-11-27

**Authors:** Monica Y Hinestroza Jordan, Karen I Salomon-Escoto, Martha Stutsky, Michael Fraher, Jonathan Kay, George Reed, Mireya Wessolossky

**Affiliations:** UMass Chan Medical School , Tucson, Massachusetts; UMass Chan Medical School, Worcester, Massachusetts; Shields Health Solutions, Bethany, Connecticut; Shields Health Solutions, Bethany, Connecticut; UMass Chan Medical School, Worcester, Massachusetts; UMass Chan Medical School, Worcester, Massachusetts; UMASS Chan Medical School, Hopkinton, Massachusetts

## Abstract

**Background:**

Patients with HIV who adhere to antiretroviral (ARV) therapy can achieve and maintain viral suppression (defined as < 200 copies of HIV/mL). Integrated Health System Specialty Pharmacies (HSSPs) help patients overcome barriers to ARV adherence, but there are limited data on the impact of HSSPs on clinical outcomes. This study compared viral suppression for patients filling ARVs at UMass Memorial Specialty Pharmacy to those utilizing a non-integrated specialty pharmacy (non-HSSP).

**Methods:**

In this retrospective cohort study, patients aged ≥18 years with an HIV diagnosis, encounter at the UMass Memorial Medical HIV Clinic, an ARV medication order, and at least one viral load (VL) result between January 2018 and May 2022 were identified from the medical record. Data included: age, sex, race, ethnicity, comorbidities, and all VL results over the study period. Time of first HSSP fill date was used to identify patient start in the respective pharmacy. VL results were divided into time under HSSP or non-HSSP (Figure 1). The index time was first VL for patients in either the HSSP or non-HSSP group.

To account for multiple VL measures per patient, a Generalized Estimating Equation logistic regression estimated odds ratios (OR) and a 95% confidence interval. An OR >1 indicates greater viral suppression.

**Figure:**
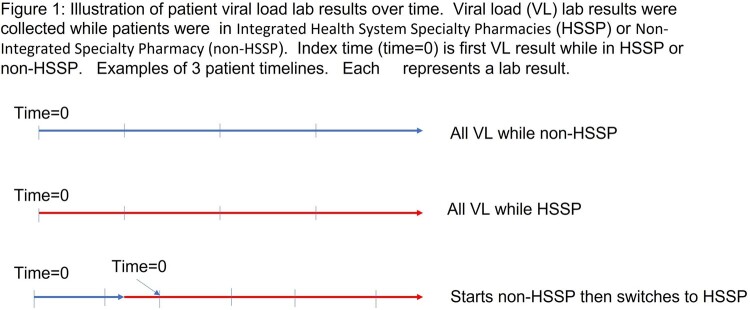

Viral load (VL) lab results were collected while patients were in Integrated Health System Specialty Pharmacies (HSSP) or Non-Integrated Specialty Pharmacy (Non-HSSP). Index time (time=0) is firts VL result while in HSSP or Non-HSSP. Examples of 3 patients timelines, each represents a lab result.

**Results:**

Of the 889 patients identified, 326 provided VL results under HSSP while 681 contributed results for non-HSSP; 118 patients provided results for both groups (Figure 2). Of the 5,295 VL results, 2,028 were from HSSP patients and 3,267 from the non-HSSP group. Of the 5,295 total VLs, 90.6% indicated viral suppression, with the average rate of 91.0% in the HSSP group vs 86.0% in the non-HSSP group. Table 1 displays unadjusted and adjusted ORs estimates. The HSSP group had a higher rate of viral suppression (adjusted OR = 1.89 95% CI: [1.40, 2.56]). Sex, ethnicity, and race were not significantly associated with viral suppression, which decreased with Charlson Comorbidity Index (CCI) 1-3; increased with age; and increased over time (from index date of VL).

**Figure:**
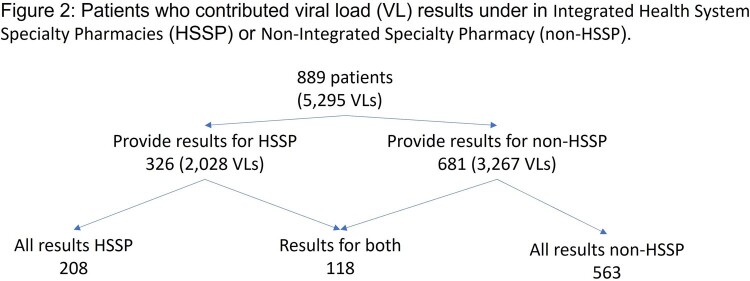

**Figure:**
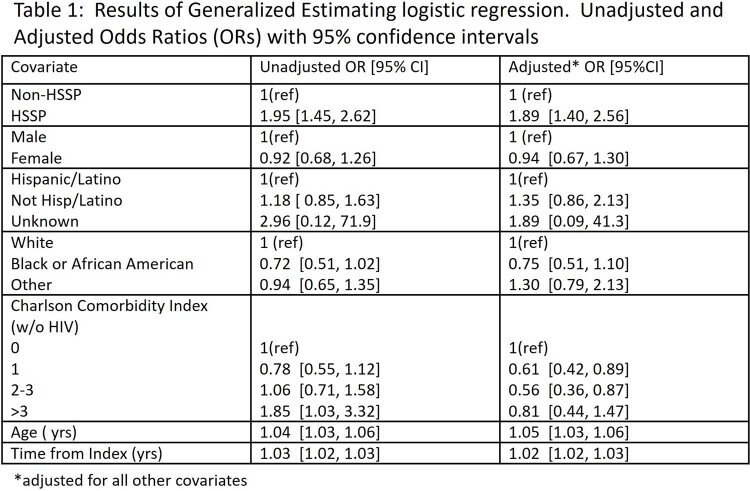

**Conclusion:**

Among patients evaluated at UMass Memorial HIV Clinic, those filling ARV medications at the HSSP demonstrated higher rates of viral suppression compared to patients utilizing a non-HSSP.

**Disclosures:**

**Karen I. Salomon-Escoto, MD**, Novartis: clinical trial PI **Jonathan Kay, MD**, Aker BioMarine AS: Grant/Research Support|Alvotech Swiss AG: Advisor/Consultant|Boehringer Ingelheim GmbH: Advisor/Consultant|Bristol Myers Squibb Co.: Advisor/Consultant|Fresenius Kabi: Advisor/Consultant|Galapagos NV: Grant/Research Support|Inmagene LLC: Independent Data Monitoring Committee member|Kolon TissueGene, Inc.: Independent Data Monitoring Committee member|Novartis Pharmaceuticals Corp.: Advisor/Consultant|Organon LLC: Advisor/Consultant|Pfizer Inc.: Advisor/Consultant|Samsung Bioepis: Advisor/Consultant|Sandoz Inc.: Advisor/Consultant|Scipher Medicine: Advisor/Consultant|Teijin Pharma Ltd.: Advisor/Consultant|Wolters Kluwer NV: Royalties for UpToDate

